# Catalase T-Deficient Fission Yeast Meiocytes Show Resistance to Ionizing Radiation

**DOI:** 10.3390/antiox9090881

**Published:** 2020-09-17

**Authors:** Razan Muhtadi, Alexander Lorenz, Samantha J. Mpaulo, Christian Siebenwirth, Harry Scherthan

**Affiliations:** 1 Institut für Radiobiologie der Bundeswehr in Verb. mit der Universität Ulm, Neuherbergstr. 11, D-80937 Munich, Germany; razan_muhtadi@gmx.de (R.M.); ChristianSiebenwirth@bundeswehr.org (C.S.); 2 Institute of Medical Sciences (IMS), University of Aberdeen, Foresterhill, Aberdeen AB25 2ZD, UK; a.lorenz@abdn.ac.uk (A.L.); s.mpaulo@abdn.ac.uk (S.J.M.)

**Keywords:** chromosome mobility, Ctt1, horsetail movement, ionizing radiation, meiosis, radical stress, live-cell microscopy, meiosis, Pcl1, ROS, *Schizosaccharomyces pombe*, sporulation

## Abstract

Environmental stress, reactive oxygen species (ROS), or ionizing radiation (IR) can induce adverse effects in organisms and their cells, including mutations and premature aging. DNA damage and its faulty repair can lead to cell death or promote cancer through the accumulation of mutations. Misrepair in germ cells is particularly dangerous as it may lead to alterations in developmental programs and genetic disease in the offspring. DNA damage pathways and radical defense mechanisms mediate resistance to genotoxic stresses. Here, we investigated, in the fission yeast *Schizosaccharomyces pombe*, the role of the H_2_O_2_-detoxifying enzyme cytosolic catalase T (Ctt1) and the Fe^2+^/Mn^2+^ symporter Pcl1 in protecting meiotic chromosome dynamics and gamete formation from radicals generated by ROS and IR. We found that wild-type and *pcl1*-deficient cells respond similarly to X ray doses of up to 300 Gy, while *ctt1*∆ meiocytes showed a moderate sensitivity to IR but a hypersensitivity to hydrogen peroxide with cells dying at >0.4 mM H_2_O_2_. Meiocytes deficient for *pcl1*, on the other hand, showed a resistance to hydrogen peroxide similar to that of the wild type, surviving doses >40 mM. In all, it appears that in the absence of the main H_2_O_2_-detoxifying pathway *S. pombe* meiocytes are able to survive significant doses of IR-induced radicals.

## 1. Introduction

Environmental stressors, such as ultraviolet light or ionizing radiation (IR), induce a plethora of cellular injuries and responses, among which DNA damage can severely threaten survival. While a cell seeks to maintain genomic integrity, faulty DNA damage repair may have dire consequences where mutations in coding and regulatory sequences can lead to cell death or cause cancer [1,2]. In germ cells, faulty DNA repair may lead to alterations in developmental programs and, among other effects, genetic disease in the offspring [3,4,5] and reduced viability of germ cells [6,7]. Therefore, it is desirable to understand cellular responses of meiotic cells to environmental stressors. 

Meiosis produces haploid cells by two successive cell divisions that lack an intervening S phase. This process depends on pairing of homologous chromosomes (homologs) as a prerequisite for their correct segregation during the meiotic divisions. Initially, homolog alignment is promoted by meiotic chromosome movements feeding into intimate homolog pairing by homologous recombination and/or synaptonemal complex formation in most species. Homologous recombination induced by programmed DNA double strand breaks (DSBs) leads to the formation of at least one exchange (chiasma) per chromosome, thereby ensuring the correct segregation of homologs in the first meiotic division. By the second meiotic division, the genome has been reduced to a haploid state (reviewed in [8]). Any perturbation of this complex process has serious consequences and may lead to infertility (reviewed in [9,10]). In most organisms, meiotic homolog search is driven by chromosome movements in prophase I in the presence of meiotic DSBs (reviewed in [11,12]). Besides physiological meiotic DSBs, exogenous DSBs (e.g., those resulting from IR) can be toxic, since their (mis)repair may lead to non-homologous or ectopic exchange events involving, at the chromosomal level, translocations and the formation of dicentric and acentric chromosome fragments [13,14,15,16]. Chromosome mobility in the first meiotic prophase is dependent on the cytoskeleton, either on microtubules (*S. pombe* [17]; mouse [18,19]) or actin (*Saccharomyces cerevisiae* [20]). Especially the actin cytoskeleton is sensitive to oxidative radical attack [21]. 

IR exposure of budding yeast (*S. cerevisiae*) meiocytes has been found to induce relatively few exogenous DSBs in comparison to meiotic DSBs [22], while it induces radical stress and reactive oxygen species (ROS) that in turn can lead to protein oxidation involving the actin cytoskeleton, thereby impairing meiotic chromosome movements [22]. Meiotic chromosome movements in the fission yeast *S. pombe*, on the other hand, depend on astral microtubule dynamics and dynein motors that drag the nucleus through the zygote; known as horsetail movements [17,23]. *S. pombe* meiocytes, and particularly their chromosome motility, have been found to be highly radioresistant, a feature that relies on a potent antioxidant response, also protecting the cytoskeleton of meiocytes [6]. However, the underlying features of this potent antioxidant defense of fission yeast meiocytes require further analysis.

Eukaryotes have two antioxidant systems, a canonical one [24,25] and an ancient Mn-dependent one. The latter is highly conserved and considered a major component of the high radioresistance of bacteria, such as *Deinococcus radiodurans* [26,27]. In budding yeast, manganese metabolites can function as antioxidants detoxifying superoxide [28]. Such Mn^2+^-complex antioxidants in high concentrations can complement for the absence of superoxide dismutase (SOD) activity, suggesting that this oxidative stress response pathway may be important for survival under environmental stress [26]. Relevant *Saccharomyces cerevisiae* genes contributing to the formation of Mn^2+^ antioxidant metabolite complexes are *ATX2*, *BSD2*, *CCC1*, and *PHO80/85* (cyclin-CDK complex controlling phosphate uptake and stress response), whose loss leads to deprivation of Mn^2+^ and thus its metabolites to function as antioxidant, rendering cells sensitive to oxygen and superoxide [26]. 

Vegetative *S. pombe* cells have a strong antioxidant defense responsive to environmental stress [29]. Since the role of Mn-dependent antioxidants in *S. pombe* has so far not been explored, we searched the databases for the presence of *S. pombe* homologs to the *Saccharomyces cerevisiae* genes noted above. We identified *S. pombe* homologs for *ATX2* and *BSD2* (negative regulators of the yeast Nramp Mn transporters), *CCC1* (vacuolar Fe^2+^/Mn^2+^ symporter), and *PHO85* (involved in CDK-dependent stress-signaling). Because, so far, unknown antioxidant components may contribute to the potent ROS resistance of *S. pombe* meiocytes, we investigated the role of the antioxidant defense for the radioresistance of *S. pombe* meiosis by knocking out the homolog of the budding yeast *CCC1* vacuolar Fe^2+^/Mn^2+^-symporter gene *pcl1*, as well as the *S. pombe* gene for the cytosolic catalase T Ctt1 (*ctt1*), the main canonical peroxide scavenger [30,31,32,33]. The latter was of interest since *Saccharomyces cerevisiae* mutants for catalase display different sensitivities to ionization radiation and to hydrogen peroxide [34]. Here we tested the response of *pcl1* and *ctt1* mutant meiocytes to IR and peroxide stress in sporulation experiments.

## 2. Materials and Methods

### 2.1. Yeast Strains and Their Construction 

*Schizosaccharomyces pombe* strains (Table 1) were cultured on yeast extract (YE) (supplements added to a final concentration of 250 µg/mL) and yeast nitrogen base glutamate agar plates (supplements at a final concentration of 75 µg/mL). Crosses were performed on malt extract agar (supplements at a final concentration of 50 µg/mL). 

*Escherichia coli* was grown in LB (Lysogeny Broth) and SOC (Super Optimal broth with Catabolite repression) media, when appropriate media contained 100 µg/mL Ampicillin. Competent *E. coli* NEB10-beta were transformed according to the protocol supplied by the manufacturer (New England Biolabs, Inc., Ipswich, MA, USA).

High-fidelity DNA polymerase Q5, restriction endonucleases, and the NEBuilder HiFi DNA Assembly Master Mix were obtained from New England Biolabs. All sequence details and positional information about *S. pombe* genomic loci have been extracted from https://www.pombase.org/. To generate deletion cassettes for *pcl1* and *ctt1* we followed a previously described strategy [38]. For the *pcl1*∆-producing construct, the backbone and the dominant drug resistance marker (*hphMX4*) of pAG32 [39] after a *Pvu*II-*Eco*RV digest were merged with a 398 bp upstream (oligonucleotides oUA600 5′-ATAGAACGCGGCCGCCAGAGCACTTATTTGTGGGC-3′ and oUA601 5′-CAGCGTACGAAGCTTCAGCGATAGTGTAGAGGTAGTGATTG-3′) and a 426 bp downstream flanking sequence (oligonucleotides oUA602 5′-CTCGAATTCATCGATGATGCCAAACGTCTAAAGAGGG-3′ and oUA603 5′-GCCACTAGTGGATCTGATCTTTCACCATCACAGTCTCG-3′) amplified by PCR from *S. pombe* genomic DNA (strain ALP1596 and ALP714, respectively) in a single NEBuilder assembly reaction; this resulted in the vector pALo243. Similarly, for the *ctt1*∆-generating cassette a 408 bp upstream (oligonucleotides oUA606 5′-ATAGAACGCGGCCGCCAGACGTTGGTAATTCTACACC-3′ and oUA607 5′-CAGCGTACGAAGCTTCAGCCTTAGACTGAGAAGATGC-3′) and a 368 bp downstream flanking sequence (oligonucleotides oUA608 5′-CTCGAATTCATCGATGATCTCATAATGCGCTTATGCG-3′ and oUA609 5′-GCCACTAGTGGATCTGATGTAATCGAATATCGTGTGTGG-3′) amplified by PCR from *S. pombe* genomic DNA (strain ALP714) were merged with the backbone and *hphMX4* cassette of pAG32 after a *Pvu*II-*Eco*RV digest; the resulting vector is pALo244. In both instances, the *Pvu*II and *Eco*RV sites were destroyed during cloning. Relevant sections of the plasmid were verified by Sanger sequencing (Eurofins Genomics Germany GmbH, Ebersberg, Germany). The deletion cassettes for *pcl1* and *ctt1* were amplified by PCR using oligonucleotides oUA612 (5′-GAGCACTTATTTGTGGGC-3′) and oUA613 (5′-CTTTCACCATCACAGTCTCG-3′) on pALo243, and oUA614 (5′-GACGTTGGTAATTCTACACC-3′) and oUA615 (5′-GTAATCGAATATCGTGTGTGG-3′) on pALo244, respectively. The resulting cassettes were transformed into strains FO652, UoA794, and UoA795 using a standard Li-acetate protocol [40].

Recombination assay strains (UoA1040, UoA1043, UoA1044, and UoA1047) were generated by crossing of UoA1038 or UoA1039 to strains containing the relevant recombination marker alleles. Spore viability by random spore analysis and meiotic recombination assays have been performed as previously described [36].

### 2.2. Cell Culture for Meiotic Time-Courses

Briefly, strains were first induced to undergo meiosis by depleting nitrogen sources from the culture medium as described previously [41]. To induce meiosis, strains were streaked on YE plates and incubated at 30 °C for four days. Three to five clearly visible colonies were chosen from the YE plates and incubated shaking in 10 mL liquid YE overnight at 30 °C.

The cell suspension was centrifuged for 4 min at 700× *g*. The cell pellet was inoculated with 20 mL presporulation medium (PM) and incubated at 30 °C for 14–16 h under vigorous shaking. Cells were harvested at a cell number of 1–2 × 10^7^ by centrifuging 700× *g* for 4 min, washed with 50 mL distilled water, resuspended, and incubated with shaking in 30 mL sporulation medium (PM-N) at 30 °C for 3 h. Experiments were continued when >70% of cells were expressing horsetail nuclei as determined by Rec8-GFP under microscopic inspection. The extent of sporulation and prophase I nuclei was assayed by DNA staining of ethanol-fixed cells with DAPI (4′,6-diamidino-2-phenylindole) as described previously [41].

### 2.3. Spot Assays

The cell density of exponentially growing yeast cultures (fully supplemented YE at 30 °C) was determined using a haemocytometer. Cell cultures were diluted in water to a concentration of 1 × 10^7^ cells/mL. Suspensions were then serially diluted in 10-fold steps to 1 × 10^4^ cells/mL, and 10 µL aliquots of each dilution were then spotted onto YES plates with and without H_2_O_2_ (Sigma-Aldrich, Deisenhofen, Germany) at the concentrations indicated in Figure S1. Plates were photographed after 3 days of incubation at 30 °C. To verify results, spot assays were repeated multiple times.

### 2.4. X Irradiation

Five milliliter aliquots of the sporulating cultures were X irradiated in tilted 15 mL falcon tubes at room temperature using a Xylon MGC 41 X-ray device (YXLON Maxishot, Hamburg, Germany) at 240 kV and 17.5 mA and a dose rate of 5.2 Gy/min. X rays were filtered with 7.0 mm beryllium and a 2.0 mm aluminum layer. The delivered dose was measured with a Duplex dosimeter (PTW, ptwdosimetry.com, Freiburg, Germany) attached to the falcon tube.

### 2.5. H_2_O_2_ Treatment

Aliquots of sporulating cultures (5 mL) were incubated in 10 mM or 40 mM H_2_O_2_ (Carl Roth, carlroth.com, Karlsruhe, Germany) dissolved in medium for 20 min at 30 °C. Cells were pelleted by a brief spin, resuspended in sporulation medium, and immediately subjected to live-cell imaging or treated for other assays. 

### 2.6. Detection of Reactive Oxygen Species

ROS in yeast cells were detected by using two different molecular probes; Dihydroethidium (DHE; Merck Biochemicals, Darmstadt, Germany) and Dihydrorhodamine 123 (DHR123; Thermo Fischer Scientific, Schwerte, Germany).

DHE is a free radical sensor that, in its reduced form, exhibits blue fluorescence in the cell. When oxidized largely by superoxide to ethidium, it obtains a red fluorescence. DHE was added to 1 mL sporulation medium to a concentration of 20 µM, followed by incubation for 20 min before IR or H_2_O_2_ treatment. Hoechst 33342 was added to the sporulation medium to give a final conc. of 0.5 µg/mL for staining the nucleus in live cells. Finally, cells were washed twice with sporulation medium and embedded in antifade solution containing 0.25 µm TetraSpecks (Thermo Fisher Scientific; diluted 1/1000) to normalize digital image recording. Images were taken using appropriate filter sets of a Zeiss Axioimager Z2 epifluorescence microscope and the ISIS imaging system (MetaSystems, Altlussheim, Germany). Cells that fluoresced red were scored as ROS-positive.

DHR123 is a non-fluorescent radical sensor in its reduced form. When oxidized to rhodamine, it obtains a green fluorescence. Cell loading with DHR123 was done as described for DHE staining, with the final concentration of DHR123 being 100 µM.

### 2.7. Live-Cell Imaging and Image Analysis 

Live-cell imaging to determine the speed of the horsetail nucleus motility was done using a 4D live-cell microscope system (FEI.com, Gräfelfing, Germany) as described in detail elsewhere [6,42].

Time lapse movies were recorded for three minutes after the respective treatment at 0.5 Hz with 200 ms exposure time. Spot tracking and speed measurements of the leading edge of the horsetail nucleus was done by quantitative image analyses of the time lapse movies using ImageJ and the plugin Manual Tracking (https://imagej.nih.gov/ij/plugins/track/track.html).

### 2.8. Statistics

**Figure 1:**
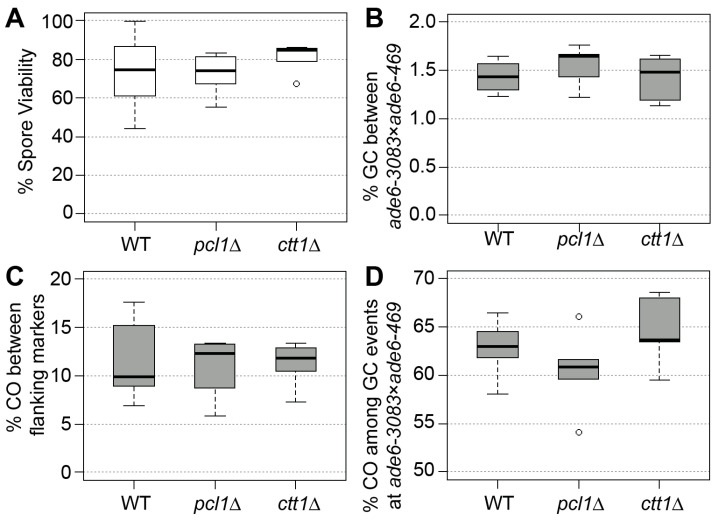
Spore viability and meiotic recombination outcome in wild type (WT), *pcl1*∆, and *ctt1*∆. (**A**) Spore viability of wild type (ALP733 × ALP731, n = 6), *pcl1*∆ (UoA1040 × UoA1043, n = 6), and *ctt1*∆ (UoA1044 × UoA1047, n = 6). (**B**) Frequency of gene conversion (GC) at *ade6-3083* × *ade6-469*, (**C**) frequency of crossovers (CO) between *his3*^+^-*aim* and *ura4*^+^-*aim2*, and (**D**) percentage of CO between *his3*^+^-*aim* and *ura4*^+^-*aim2* associated with a GC event at *ade6* in wild type and mutant crosses; strains and n as in (**A**). n indicates the number of independent crosses. The upper, middle, and lower lines of the box represent the third, second, and first quartile, respectively (second quartile = median). The ‘whiskers’ represent the minimum and maximum of the range, unless they differ more than 1.5-times the interquartile distance from the median; then, the 1.5-times interquartile distance around the median is indicated by the ‘whiskers’ and outliers, if present, which are shown as open circles.

Simple descriptive statistics including Gaussian error propagation were computed using Microsoft^®^ Office Excel (Student’s *t*-test). ANOVA analyses were performed using PRISM (graphpad.com), except for recombination data (Figure 1), which were treated as described previously [37]. A *p* value of less than 0.05 was considered statistically significant. 

## 3. Results

### 3.1. Mutants of ctt1 and pcl1 Show Normal Spore Viability or Meiotic Recombination in a Standard Environment

To check whether the deletion of the antioxidant defense gene *ctt1* or the Fe^2+^/Mn^2+^-transporter gene *pcl1* contribute to the fidelity of meiosis under standard laboratory conditions, we measured spore viability and recombination frequencies in wild type and the two deletion mutants. We employed a genetic recombination assay that allows us to determine gene conversion and crossover frequencies alongside crossover rate associated with gene conversion events [35,43]. As part of these experiments spore viability was measured, as well. Neither the absence of *ctt1* nor of *pcl1* caused a significant change (Mann–Whitney U test) in any of the recombination outcomes or the spore viability in comparison to the wild type (Figure 1). This demonstrates that the presence of Ctt1 and Pcl1 is not required for meiotic functions in a standard laboratory environment.

### 3.2. X Irradiation and Sporulation in ctt1*∆* and pcl1*∆* Strains 

**Figure 2:**
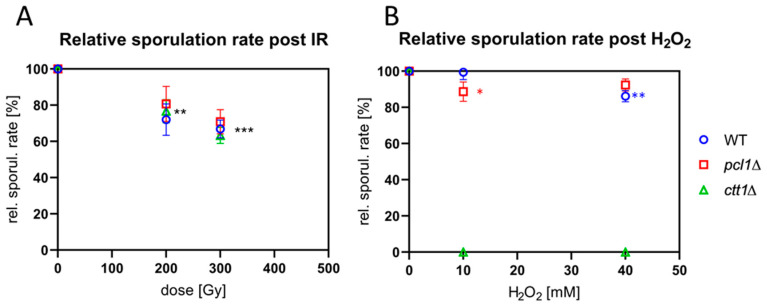
(**A**) X irradiation with 200 and 300 Gy significantly reduced the relative sporulation rate 20 h post-ionizing radiation (IR) relative to the respective non-IR control (** *p* ≤ 0.0013 and *** *p* < 0.0001, respectively; ANOVA). (**B**) Wild-type (WT) cells were resistant to 10mM H_2_O_2_ treatment, while 40 mM significantly (** *p* = 0.0012) reduced the average sporulation relative to sham-treated control. The average reduction of sporulation rate was significant for *pcl1*∆ at 10mM (* *p* = 0.011), while it was not significant post 40 mM H_2_O_2_. In contrast, all H_2_O_2_ treatments caused the death of the *ctt1*∆ meiocytes.

To determine the response of *S. pombe* meiocytes lacking *ctt1* and *pcl1* to IR, we exposed meiocytes of wild type and the two mutant strains to 240 kV X-irradiation and measured sporulation rates. Cell cultures were transferred to sporulation medium and irradiated with 200 and 300 Gy (20 and 30 krad, respectively) X rays 3–4 h after induction, when most cells (>70%) were in the horsetail stage (prophase I). Eventually, sporulation rates were determined 20 h post-IR in at least three independent experiments. X irradiation with 200 and 300 Gy significantly (*p* ≤ 0.0013 and *p* < 0.0001, resp.; ANOVA) reduced the average sporulation rate of the three strains to a similar extent, compared to the non-IR control (Figure 2A). For the wild-type strain 300 Gy of IR reduced sporulation to 67% (±4.94); for *pcl1*∆ 71% (±6.64) and *ctt1*∆ 63% (±4.44) (Figure 2A), with the IR-responses being similar. Hence, it can be concluded that absence of the catalase Ctt1 or the Fe^2+^/Mn^2+^-transporter Pcl1 does not increase radiation sensitivity relative to the wild type. 

### 3.3. ctt1∆ Meiocytes are Highly Sensitive to ROS

To investigate the sensitivity of the meiocytes of the mutant strains to ROS, we next exposed cells to different concentrations of H_2_O_2_ and determined sporulation rate. Sporulating cultures were treated with H_2_O_2_ 3–4 h after sporulation induction when most cells were in the horsetail stage. Cells were then analyzed 20 h after a sham or H_2_O_2_ treatment. Treatment of the wild type with 10 mM H_2_O_2_ showed only a negligible reduction of the average sporulation rate, while 40 mM H_2_O_2_ reduced sporulation by 12%, a significant difference (*p* = 0.0012) (Figure 2B).

Cells lacking the *pcl1* Fe^2+^/Mn^2+^-transporter gene showed 9% reduction of the average sporulation rate after exposure to 10 mM H_2_O_2_, a significant difference (*p* = 0.011; Figure 2B), while 6% sporulation reduction after 40 mM H_2_O_2_ was not significant compared to the control. Interestingly, the *pcl1*∆ strain was indistinguishable from wild type regarding its vegetative growth in the presence of H_2_O_2_ (Figure S1A,B). Overall, the sensitivity of sporulating wild-type and *pcl1*∆ meiocytes to H_2_O_2_ is rather mild (Figure 2B). 

In contrast, *ctt1*∆ meiocytes and vegetative cells were highly sensitive to H_2_O_2_-mediated ROS and died at H_2_O_2_ concentrations > 0.4 mM (Figure 2B and Figure S1). Moreover, vegetatively growing *ctt1*∆ cells showed the expected strong sensitivity to H_2_O_2_ at low concentrations (Figure S1C), corroborating Ctt1′s role as the major cytoplasmic H_2_O_2_ detoxification enzyme in fission yeast [44]. 

### 3.4. Ctt1-Deficient Cells Display High Levels of ROS

To test the levels of endogenous and induced ROS in sporulating cells, we made use of the blue ROS probe Dihydroethidium (DHE) [45,46]. Sporulating cells were loaded with the probe, which was preferentially oxidized to red ethidium by superoxide radicals; however, other ROS and reactive nitrogen species also can induce DHE oxidation [46]. We also applied the ROS probe Dihydrorhodamine 123 (DHR), which detects a number of ROS species, such as hydrogen peroxide hypochlorous acid and peroxynitrite anion ONOO^-^ [47]. To this end, sporulating cells were exposed to IR or H_2_O_2_ and then stained with 20 µM DHE or 100 µm DHR.

**Figure 3:**
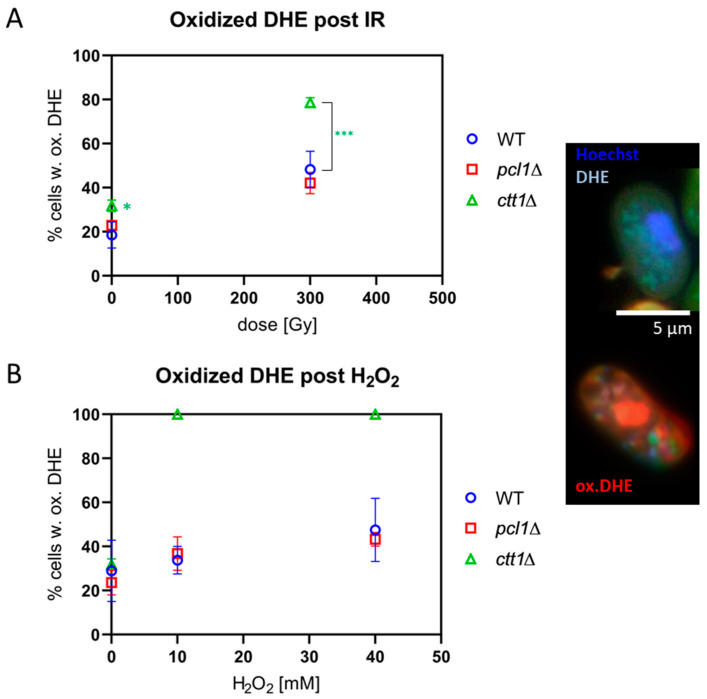
Formation of reactive oxygen species (ROS). ROS (preferentially superoxide) oxidize blue dihydroethidium to red ethidium (ox.DHE) as seen in the cells displayed to the right. Bar = 5 µm. (**A**) Among the controls *ctt1*∆ cells showed a marginally significant elevated percentage of ox.DHE-positive cells (* *p* = 0.049). After 300 Gy, X ray-exposed WT and *pcl1*∆ meiocytes showed a significant increase in the average percentage cells with oxidized DHE (*p* < 0.007) relative to sham control. For *ctt1*∆ meiocytes 300 Gy X IR induced an insignificant increase of the average percentage of ox. DHE meiocytes over sham, however, this increase at was highly significant (*** *p* < 0.0001) relative to IR-exposed WT and *pcl1*∆ meiocytes. (**B**) H_2_O_2_-induced ROS similarly increased the average percentage of ox.DHE-positive meiocytes in WT and *pcl1*∆. *ctt1*∆ cells, on the other hand, were killed by H_2_O_2_, and all showed a positive signal for ox.DHE. The differences among the controls were not significant. Data points reflect the mean of three experiments (±SD).

DHE loading of non-exposed meiocytes revealed ROS in ~20% of wild-type and *pcl1*∆ cells, and in ~30% of *ctt1*∆ cells, a marginally significant difference (*p* = 0.049) (Figure 3A). The exposure to 300 Gy X irradiation induced a highly significant (*p* ≤ 0.007) increase in meiocytes displaying oxidized DHE relative to control (Figure 3A), thus revealing IR-induced generation of superoxide and other ROS radicals in all strains tested. This increase was significantly higher in *ctt1*∆ meiocytes (*p* < 0.0001) relative to 300 Gy-exposed *pcl1*∆ and wild-type meiocytes (Figure 3A), indicating reduced scavenging of IR-induced radicals in *ctt1*∆ cells, while *pcl1*∆ cells displayed a high wild-type-like tolerance. 

**Figure 4:**
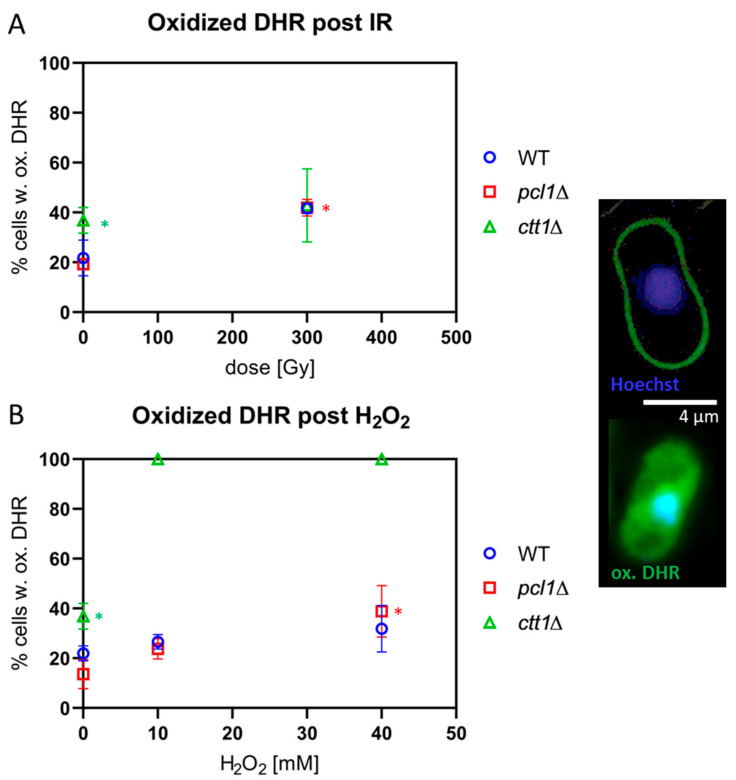
ROS formation as revealed by Dihydrorhodamine (DHR) staining. Images displayed to the right show a negative (upper cell) and a cell positive for oxidized DHR (ox.DHR; green) are. Bar = 4 µm. (**A**) Meiocytes exposed to 300 Gy of IR displayed similar average levels of ROS as indicated by ox.DHR in wild-type (WT), *pcl1*∆, and *ctt1*∆ strains. Among the non-exposed cells *ctt1*∆ meiocytes displayed the highest fraction of ox.DHR-positive cells, a significant difference (* *p* = 0.001). IR induced an increase of the percentage of ox.DHR-positive cells that is significant at 300 Gy for *pcl1*∆ and WT cells (* *p* < 0.05), while it is not significant for *ctt1*∆ meiocytes, because of the variable staining results with this mutant. (**B**) Exposure of meiocytes to H_2_O_2_ induced an increase in the average percentage of ox.DHR-positive meiocytes in WT and *pcl1*∆ relative to their control, which was significant for *pcl1*∆ at 40 mM (*p* = 0.0034). H_2_O_2_ treatment killed all *ctt1*∆ meiocytes that all were positive for ox.DHR. Data points represent the average of 3 independent experiments ± SD.

Loading sporulating cells with the ROS-sensitive probe DHR rendered similar results, with *ctt1*∆ meiocytes showing significantly more endogenous ROS-positive cells in the control relative to wild-type and *pcl1*∆ meiocytes (*p* = 0.001; Figure 4A). X-irradiation with 300 Gy induced a significant (*p* < 0.05) doubling of oxidized DHR-positive cells in wild-type and *pcl1*∆ cultures, while, in 300 Gy-exposed *ctt1*∆ cells, the average increase of oxidized DHR-positive cells was only 20% (Figure 4A); this not significant difference largely owes to the high background rate of ROS in non-exposed *ctt1*∆ meiocytes and the variable staining results in these cells. 

Next, we tested the H_2_O_2_ sensitivity of the different strains by exposure to 10 mM and 40 mM H_2_O_2_. All *ctt1*∆ meiocytes showed a full-blown DHE (Figure 3B) and DHR (Figure 4B) ROS signal after exposure to ≥10 mM H_2_O_2_, agreeing with the strong ROS formation in the absence of catalase function, eventually leading to the cell death of the treated *ctt1*∆ cells. In contrast, wild-type and *pcl1*∆ strains displayed a moderate increase of cells positive for ROS staining with growing concentrations of H_2_O_2_ peaking at an average of ~45% DHE-positive meiocytes (Figure 3B), which is not significant relative to the respective control. DHR staining showed a similar picture with exposed wild-type and *pcl1*∆ cells showing a mild increase that was significant for *pcl1*∆ at 40 mM (*p* = 0.0034) relative to control. H_2_O_2_ exposure, on the other hand, killed *ctt1*∆ meiocytes that displayed full DHR (Figure 4B) or DHE oxidation (Figure 3B) indicating formation of various ROS species by this treatment [46]. These results also agree with catalase T being the major H_2_O_2_-decomposition function in *S. pombe* [44]. 

### 3.5. Meiotic Chromosome Motility after X Irradiation

A key event in first meiotic prophase is the mobility and pairing of homologs and their telomeres [12]. After entry into prophase I, fission yeast cells attach their telomeres to the spindle pole body and perform continuous chromosome/nuclear movements that depend on astral microtubule dynamics and dynein motors that drag the nucleus through the zygote, known as horsetail movement [17,48]. Since the horsetail movement of *S. pombe* is highly radioresistant [6], we investigated whether absence of *pcl1* or *ctt1* has consequences for meiotic chromosome motility. To this end, we performed live-cell imaging of irradiated diploid wild-type and mutant *S. pombe* meiocytes that express both meiosis-specific Rec8-GFP [49] and tubulin-GFP (Atb2-GFP [50]) in the same cell. Rec8 labels the meiotic nucleus and allows for the identification of meiotic horsetail cells [6,51].

**Figure 5:**
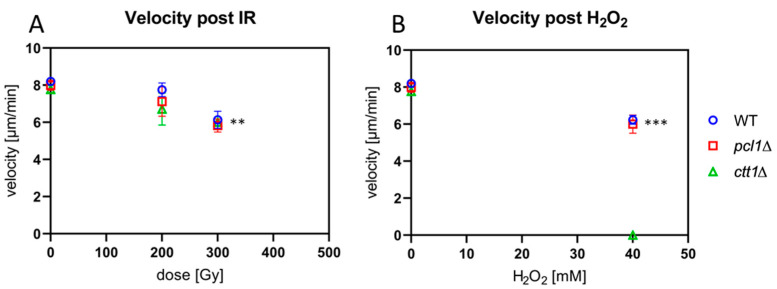
The effect of ROS stress on meiotic chromosome motility. (**A**) Exposure to 300 Gy X-IR reduced the average velocity of horsetail nuclei in wild-type, *pcl1*∆ and *ctt1*∆ strains significantly (** *p* ≤ 0.005) relative to sham-irradiated control. (**B**) H_2_O_2_ treatment (40 mM) significantly reduced average horsetail speed for the WT and *pcl1*∆ strains (*** *p* = 0.0003, *p* = 0.0002, respectively), relative to non-exposed control. H_2_O_2_ treatment killed *ctt1*∆ meiocytes. Data points reflect the mean of three experiments (± SD).

First, we measured horsetail velocity in time-lapse movies recorded from sham-irradiated and cells exposed to IR (n = 20–30 cells per exposure condition in each of three independent repeat experiments). Quantitative image analysis of the movements of the leading edge (spindle pole body) of non-exposed horsetail nuclei revealed a similar average horsetail speed of 8.2 µm/min (±0.11), 7.99 µm/min (±0.27) and 7.77 (±0.21) µm/min for the wild-type, *pcl1*∆, and *ctt1*∆ strains, respectively (Figure 5A).

Irradiation with 300 Gy X-rays led to a reduction of the average speed from ~8 µm/min in the non-exposed cells down to 6.14 µm/min, 5.82 µm/min, and 6.00 µm/min for the wild-type, *pcl1*∆, and *ctt1*∆ strains, respectively (Figure 5A), with the reductions being statistically significant relative to control (*p* ≤ 0.005). The differences between the three strains, however, were not significant. Thus, it appears that the strains tested display a similar horsetail motility response to the exposure with low linear energy transfer (LET) ionizing radiation and that the absence of catalase T is not sensitizing the meiotic chromosome movements and the microtubule cytoskeleton to IR exposure.

### 3.6. Peroxide Stress Slows Meiotic Horsetail Movements

Next, we tested the peroxide sensitivity of meiotic chromosome movements in our strains. Treating wild-type meiocytes with 10 mM H_2_O_2_ did not change the average speed of horsetail movement (8.20 µm/min vs 8.17 µm/min) (not shown). Treatment with 40 mM H_2_O_2_ exposure reduced the average wild-type horsetail motility significantly (*p* = 0.0003) to 6.24 µm/min. In the *pcl1*∆ mutant motility was similarly reduced to 6.01 µm/min (*p* = 0.0002) (Figure 5B). In contrast, all the H_2_O_2_-treated *ctt1*∆ meiocytes died at the concentrations used (Figure 5B). It appears that wild-type and *pcl1*∆ meiocytes are resistant to high levels of H_2_O_2_, while the *ctt1*∆ mutant showed the expected sensitivity. The latter contrasts with its relative resistance against IR-induced ROS (Figure 5B), which may be related to IR mostly inducing hydroxyl radicals and hydrated electrons, while H_2_O_2_ is the major target of catalase T decomposition [44].

### 3.7. Radical Stress Slows Prophase I Progression

**Figure 6:**
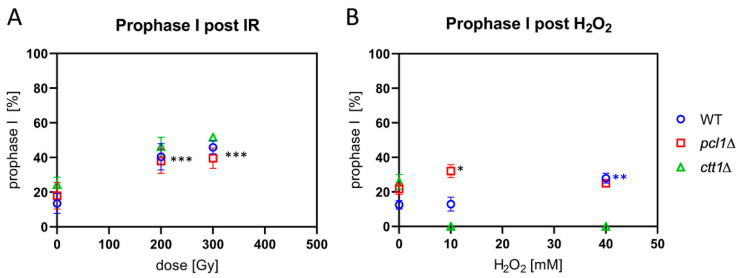
Prophase I progression after IR and H_2_O_2_ exposure. (**A**) IR significantly increased the average percentage of horsetail cells in prophase I in wild-type (WT), *pcl1*∆, and *ctt1*∆ strains at both doses used (*** p ≤ 0.0005), relative to control. (**B**) H_2_O_2_ treatment killed *ctt1*∆ meiocytes, but significantly (* *p* = 0.013) increased the average percentage of *pcl1*∆ horsetail prophase I cells relative to control and 10 mM-treated WT cells. 40 mM H_2_O_2_ led to a significant increase of horsetail cells only in the WT (** p = 0.0006), indicating delayed progression through WT prophase I. Data points reflect the mean of three experiments (±SD).

The progression of fission yeast cells through prophase I can be retarded by IR or ROS exposure [6]. Therefore, we tested prophase I progression in the *pcl1*∆ and the peroxide-sensitive *ctt1*∆ mutant by irradiating sporulating cultures with X rays. It appeared that exposure to 200 and 300 Gy IR induced a significant (*p* ≤ 0.0005) delay in prophase I progression, with a two-fold increase in the average frequency of horsetail nuclei in irradiated wild-type, *pcl1*∆, and *ctt1*∆ strains (Figure 6A).

Exposing wild-type cells to 10 mM H_2_O_2_, on the other hand, failed to induce a delay in prophase I, while there were more cells in first meiotic prophase after 40 mM H_2_O_2_ treatment (Figure 6B), a significant difference (*p* = 0.0006). This indicates a delayed progression through wild-type prophase I under peroxide stress. H_2_O_2_-treated *pcl1*∆ meiocytes showed a delay in prophase I progression at both H_2_O_2_ concentrations tested (Figure 6B), with only the increase after 10 mM H_2_O_2_ treatment being statistically significant (*p* = 0.013). The significant increase at 10 mM interestingly matches the slight but significantly reduced sporulation rate at 10 mM in the *pcl1*∆ mutant (Figure 2B), indicating a hypersensitivity at lower doses, which could indicate a role of Mn in mediating ROS protection at low doses. Sporulating *ctt1*∆ meiocytes were readily killed by H_2_O_2_ exposure (Figure 6B), again confirming the strong sensitivity of catalase T-deficient cells to H_2_O_2_.

### 3.8. IR and ROS Exposure Induces Aberrant Ascospores

Since IR and ROS induce DNA double strand breaks in which misrepair can lead to chromosome aberrations and micronucleus formation after cell division, we analyzed asci for the presence of aberrant numbers of nuclei, which are indicative of chromosome fragments, missegregation and genome instability. Sporulating cultures were irradiated with 200 Gy and 300 Gy or treated with 10 mM and 40 mM H_2_O_2_ and the number of DAPI-stained spore nuclei enumerated in asci. Asci with more or less than 4 spore nuclei were scored as aberrant in three independent experiments.

**Figure 7:**
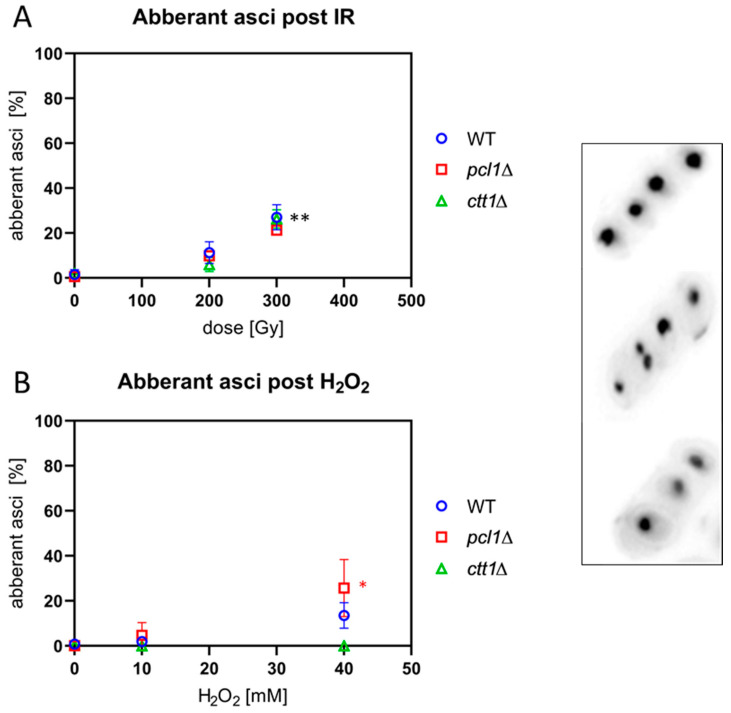
IR and ROS exposure induce aberrant asci, as shown in microscopic images on the right. The upper ascus contains 4 normal ascospores, while the two aberrant asci below show 5 and 3 spore nuclei, respectively. (**A**) X irradiation significantly increased the average frequency of aberrant asci in wild-type (WT), *pcl1*∆, and *ctt1*∆ strains at 300 Gy (** *p* < 0.007) relative to sham IR. (**B**) H_2_O_2_ exposure induced a not significant increase in the WT but a significant increase of aberrant asci in *pcl1*∆ at 40 mM (* *p* = 0.025). *ctt1*∆ meiocytes were killed by H_2_O_2_, blocking spore formation. Data points reflect the mean of three experiments (± SD).

Exposure to 200 Gy X-irradiation induced a slight increase in the average percentage of aberrant asci in all three strains that became significant at 300 Gy (*p* ≤ 0.007) relative to the respective non-irradiated control (Figure 7A).

When the strains were exposed to peroxide, we observed the expected hypersensitivity of *ctt1*∆ meiocytes leading to cell death at 10 mM H_2_O_2_ and above (Figure 7B). In the wild type, 10 mM H_2_O_2_ treatment failed to induce aberrant asci, while there was an insignificant increase after 40 mM H_2_O_2_. Exposed *pcl1*∆ cells showed a significant increase of the average number of aberrant asci after treatment with 40 mM (*p* = 0.025) H_2_O_2_ (Figure 7B). The significant increase of aberrant *pcl1*∆ asci after high H_2_O_2_ treatment, absent in the wild type, may point to a role of Mn^2+^ metabolism in mediating some protection from ROS in fission yeast.

## 4. Discussion

Meiosis is the central mechanism of sexual reproduction and produces haploid cells by two successive cell divisions without an intervening S phase. Meiosis involves formation of programmed DSBs whose repair by homologous recombination allows for accurate homolog segregation and eventually healthy haploid gametes or spores (for a review see [8]). In most organisms, meiotic chromosome movements in the presence of meiotic DSBs contributes to homolog recognition and alignment and thus to the fidelity of meiosis (for review see [11,12]). Exposure of meiocytes to genotoxic agents (e.g., IR, ROS) can lead to cell death or increase of genomic damage and mutations [52].

Only few exogenous DSBs are induced by exposing budding yeast meiocytes to IR, far fewer DSBs than would normally occur during meiosis. Importantly, IR rather generates radical stress that leads to protein oxidation involving the actin cytoskeleton, thus compromising meiotic chromosome movements [22]. In contrast, meiotic chromosome movements in the fission yeast *S. pombe*, known as horsetail movements, are microtubule-dependent [17]. *S. pombe* meiocytes and horsetail movements are highly radioresistant, a feature that relies on a potent antioxidant response [6]. Since the underlying features of this potent antioxidant defense of fission yeast meiocytes remain to be explored, we studied the IR and H_2_O_2_ response of meiocytes lacking the cytosolic catalase T (Ctt1) that is the main H_2_O_2_-detoxifying enzyme of *S. pombe* [30], as well as of a strain deleted for the vacuolar Fe^2+^/Mn^2+^-transporter gene *pcl1*. The latter was of interest, since Mn^2+^ complex antioxidants have been considered to contribute significantly to the high resistance of bacteria and budding yeast to IR and oxygen stress [26,28].

In all the endpoints studied after ionizing radiation and H_2_O_2_ exposure, *pcl1*∆ meiocytes behaved like wild type except for a 10 mM H_2_O_2_ exposure leading to less efficient sporulation and a slowed progression through prophase I as indicated by an increased percentage of meiotic prophase cells still in the horsetail stage 20 h post-exposure. While this may point to low dose hypersensitivity in the *pcl1* mutant, this effect was absent after a 40 mM H_2_O_2_ challenge. Hence, we conclude that the absence of Pcl1 in meiocytes is not significantly contributing to the strong antioxidant defense system protecting meiotic *S. pombe* cells. This agrees with chromosome mobility of *pcl1*∆ and wild-type meiocytes being similarly responsive to H_2_O_2_. However, further experiments are needed to address whether there may be a role for other Mn-dependent functions in protecting *S. pombe* cells from ROS.

In contrast, deletion of the cytosolic catalase T gene (*ctt1)* of *S. pombe* induced a high sensitivity of the mutant strain to treatment with peroxide concentrations still tolerated by wild-type and *pcl1*∆ cells. While prophase I progression in the wild-type and *pcl1*∆ strains was hardly retarded by 40 mM H_2_O_2_ treatment of the sporulating cultures, *ctt1*∆ cells were killed at peroxide concentrations above 0.2 mM. This is in agreement with earlier reports showing that Ctt1 is the main H_2_O_2_-scavenging enzyme of *S. pombe* addressing high levels of peroxide [30,33,44].

Staining with the superoxide-probe DHE and ROS-probe DHR showed high levels of the ROS indicators in unexposed *ctt1*∆ meiocytes showing elevated endogenous ROS in untreated cells without catalase T. Exposure with H_2_O_2_ induced ROS signals in all treated *ctt1*∆ meiocytes. Wild-type and *pcl1*∆ cells, in contrast, showed only a 1.6-fold increase of oxidized DHE-positive cells after 40 mM H_2_O_2_ treatment, indicating that peroxide detoxification is largely intact in those cells. DHR staining, which reacts with various peroxides, revealed similar results, in agreement with DHE being capable to form microscopic signals with oxidants other than superoxide [46].

Exposure to ionizing radiation, in contrast, induced only a moderate response in catalase-deficient cells, with 300 Gy of X irradiation reducing sporulation of *ctt1*∆ cells similar to irradiated wild-type and *pcl1*∆ meiocytes. Similar results were obtained for the responsiveness of meiotic chromosome mobility that was similarly reduced in *ctt1*∆, *pcl1*∆, and wild-type meiocytes after 300 Gy X ray exposure, as was the formation of aberrant asci as a measure for chromosome fragmentation, misrepair and missegregation. IR of the three strains also induced a similar increase in cells in prophase I, revealing the expected retardation of prophase I progression after irradiation, observed in *S. pombe* and *Saccharomyces cerevisiae* meiosis before [6,22].

The above results stipulate that the peroxide detoxification function of catalase T is not a major factor conferring radioresistance in *S. pombe* meiocytes. The reason for the different sensitivity of *ctt1*∆ cells to IR and H_2_O_2_ exposure may lie in the nature of radicals induced and the different responses addressing them [31]. While low endogenous levels of peroxides can be scavenged by peroxiredoxin Tpx1, Catalase T is the major decomposition enzyme for peroxides in *S. pombe* cells at high H_2_O_2_ concentrations [32,33,44], matching the situation in *Saccharomyces cerevisiae* [53]. On the other hand, IR largely produces hydroxyl and hydrogen radicals, hydrated e^-^, superoxide, and H_2_O_2_ from radiolysis of water [52,54]. It is thus very likely that the remaining antioxidant systems in *ctt1*∆ cells like superoxide dismutases or the glutathione system [31,55] can still scavenge ∙OH radicals allowing them to survive IR exposures, while still being extremely sensitive to high peroxide concentrations. Interestingly, Nishimoto and coworkers observed that vegetatively growing budding yeast *ctt1* mutant cells are hypersensitive to H_2_O_2_ exposure but also relatively resistant to IR, especially in stationary phase, which led the authors to conclude that other factors contribute to the tolerance of *ctt1*∆ cells against radicals induced by irradiation [34,53]. In all, our results are in agreement with catalase T being the major detoxification enzyme of high H_2_O_2_ levels in fission yeast [32,33,44], while this ROS-protective function seems to be of minor importance for radioresistance. Thus, further analyses of proteins like Sty1 or Pap1 in the antioxidant defense of *S. pombe* [29] will have to further elucidate the potent radioresistance of fission yeast meiocytes.

## Figures and Tables

**Table 1:** *Schizosaccharomyces pombe* strains and their genotype.

Strain Name	Genotype	Origin/Source
ALP714	*h^+S^*	[35]
ALP731	*h^-smt0^ ade6-469 his3^+^-aim arg3-D4 his3-D1 ura4-D18*	[36]
ALP733	*h^+S^ ade6-3083 ura4^+^-aim2 his3-D1 leu1-32 ura4-D18*	[36]
ALP1596	*h^-smt0^ ade7-152 his3-D1 leu1-32 ura4-D18*	[37]
FO652	*h^-smt0^ arg3-D4 his3-D1 leu1-32 ura4-D18*	[37]
UoA794 ^a^	*h^+S^ GFP-atb2*^+^::*natMX4 rec8*^+^::*GFP*-*kanMX6 ade6-M210 his1-102 leu1-32*	this study
UoA795 ^a^	*h^-smt0^ GFP-atb2*^+^::*natMX4 rec8*^+^::*GFP*-*kanMX6 ade6-M216 leu2-120 his7-366*	this study
UoA798 (WT)	*h^+S^*/*h^-smt0^ GFP-atb2*^+^::*natMX4*/*GFP-atb2*^+^::*natMX4 rec8*^+^::*GFP*-*kanMX6*/*rec8*^+^::*GFP-kanMX6 ade6-M210*/*ade6-M216 his1-102*/*his1*^+^* leu2-120*/*leu2*^+^* leu1-32*/*leu1*^+^* his7-366*/*his7*^+^	this study; cross of UoA794 × UoA795
UoA992	*h^+S^ pcl1*∆*-21a*::*hphMX4 GFP-atb2*^+^::*natMX4 rec8*^+^::*GFP-kanMX6 ade6-M210 his1-102 leu1-32*	this study; derivative of UoA794
UoA993	*h^-smt0^ pcl1*∆*-21b*::*hphMX4 GFP-atb2*^+^::*natMX4 rec8*^+^::*GFP-kanMX6 ade6-M216 leu2-120 his7-366*	this study; derivative of UoA795
UoA994 (*pcl1*∆)	*h^+S^*/*h^-smt0^ pcl1*∆*-21a*::*hphMX4*/*pcl1*∆*-21b*::*hphMX4 GFP-atb2*^+^::*natMX4*/*GFP-atb2*^+^::*natMX4 rec8*^+^::*GFP*-*kanMX6*/*rec8*^+^::*GFP-kanMX6 ade6-M210*/*ade6-M216 his1-102*/*his1*^+^* leu2-120*/*leu2*^+^* leu1-32*/*leu1*^+^* his7-366*/*his7*^+^	this study; cross of UoA992 × UoA993
UoA995	*h^+S^ ctt1*∆*-18a*::*hphMX4 GFP-atb2*^+^::*natMX4 rec8*^+^::*GFP-kanMX6 ade6-M210 his1-102 leu1-32*	this study; derivative of UoA794
UoA996	*h^-smt0^ ctt1*∆-*18b*::*hphMX4 GFP-atb2*^+^::*natMX4 rec8*^+^::*GFP-kanMX6 ade6-M216 leu2-120 his7-366*	this study; derivative of UoA795
UoA997 (*ctt1*∆)	*h^+S^*/*h^-smt0^ ctt1*∆*-18a*::*hphMX4*/*ctt1*∆-*18b*::*hphMX4 GFP-atb2*^+^::*natMX4*/*GFP-atb2*^+^::*natMX4 rec8*^+^::*GFP*-*kanMX6*/*rec8*^+^::*GFP-kanMX6 ade6-M210*/*ade6-M216 his1-102*/*his1*^+^* leu2-120*/*leu2*^+^* leu1-32*/*leu1*^+^* his7-366*/*his7*^+^	this study; cross of UoA995 × UoA996
UoA1038	*h^-smt0^ pcl1*∆*-21c*::*hphMX4 arg3-D4 his3-D1 leu1-32 ura4-D18*	this study; derivative of FO652
UoA1039	*h^-smt0^ ctt1*∆*-18c*::*hphMX4 arg3-D4 his3-D1 leu1-32 ura4-D18*	this study; derivative of FO652
UoA1040	*h^+S^ pcl1*∆*-21c*::*hphMX4 ade6-3083 ura4^+^-aim2 his3-D1 leu1-32 ura4-D18*	this study
UoA1043	*h^-smt0^ pcl1*∆*-21c*::*hphMX4 ade6-469 his3^+^-aim arg3-D4 his3-D1 ura4-D18*	this study
UoA1044	*h^+S^ ctt1*∆*-18c*::*hphMX4 ade6-3083 ura4^+^-aim2 his3-D1 leu1-32 ura4-D18*	this study
UoA1047	*h^-smt0^ ctt1*∆*-18c*::*hphMX4 ade6-469 his3^+^-aim arg3-D4 his3-D1 ura4-D18*	this study

^a^*GFP-atb2*^+^::*natMX4* strains are derivatives of MS1428 (FY17687) provided by the National BioResource Project (NBRP) of the MEXT, Japan.

## Supplementary Materials

The following are available online at https://www.mdpi.com/2076-3921/9/9/881/s1, Figure S1: Spot assays.
