# Supplementary material for: Catalase T-Deficient Fission Yeast Meiocytes Show Resistance to Ionizing Radiation
Source: Antioxidants (Basel). 2020 Sep 17;9(9):881. doi: 10.3390/antiox9090881 (PMC7555645; doi:10.3390/antiox9090881)
Supplement: Supplementary file 1 [file antioxidants-09-00881-s001.pdf]

Suppl. Fig. S1

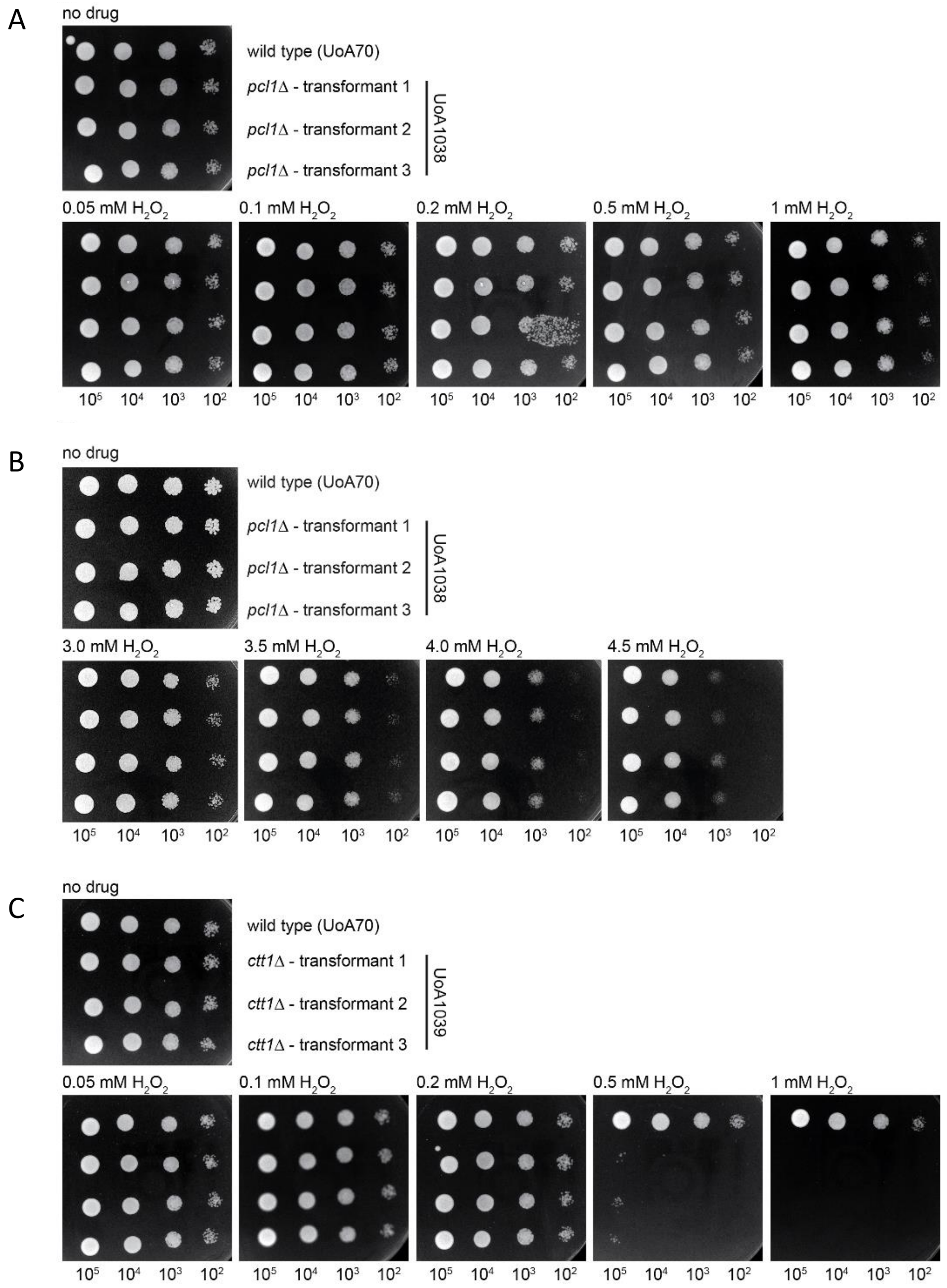

Supplementary Figure S1: **Spot assays** of wild-type (UoA70), *pcl1*Δ (UoA1038), and *ctt1*Δ (UoA1039) mutant strains on solid growth media containing the indicated concentration of H_2O_2.
